# Heterologous expression of hyperthermophilic cellulases of archaea *Pyrococcus* sp. by fungus *Talaromyces cellulolyticus*

**DOI:** 10.1007/s10295-014-1532-2

**Published:** 2014-11-12

**Authors:** Seiichiro Kishishita, Tatsuya Fujii, Kazuhiko Ishikawa

**Affiliations:** Biomass Refinery Research Center, National Institute of Advanced Industrial Science and Technology, 3-11-32 Kagamiyama, Higashi-Hiroshima, Hiroshima 739-0046 Japan

**Keywords:** Biomass, Cellulase, Fungus, Archaea, Production

## Abstract

*Talaromyces cellulolyticus* (formerly known as *Acremonium cellulolyticus*) is one of the high cellulolytic enzyme-producing fungi. *T. cellulolyticus* exhibits the potential ability for high amount production of enzyme proteins. Using the homologous expression system under the control of a glucoamylase promoter, some kinds of cellulases of *T. cellulolyticus* can be expressed by *T. cellulolyticus*. On the other hand, hyperthermophilic cellulase has been expected to be useful in the industrial applications to biomass. The hyperthermophilic archaea *Pyrococcus horikoshii* and *P. furiosus* have GH family 5 and 12 hyperthermophilic endocellulase, respectively. The two kinds of hyperthermophilic endocellulases were successfully produced by *T. cellulolyticus* using the above expression system under the control of a glucoamylase promoter of *T. cellulolyticus*. These recombinant cellulases exhibited the same characteristics as those of the recombinant cellulases prepared in *E. coli.* The productions of the recombinant enzymes were estimated to be over 100 mg/L. In this study, we first report the overexpression of the hyperthermophilic enzymes of archaea using the fungal expression system.

## Introduction

Cellulase is one of the most important industrial enzyme in terms of biomass utilization, since the enzyme has a key role to play in the degradation of β-glucan cellulose. Recent research for biofuel production from lignocellulose biomass has been allowed to accelerate development of an ideal cellulase for efficient biomass saccharification. Among them, hyperthermophilic cellulase would be very useful in industrial applications, because enzymatic reaction process at high temperature has many merits such as reducing risk of microbial contamination, increasing of the solubility of substrates and improving reaction rate. Therefore, many researchers have been focusing on the development of thermophilic cellulase with high activity. Some of hyperthermophilic β-1,4 endocellulases (endo-type cellulase) have been found in the genome database of several hyperthermophilic archaea. The hyperthermophilic archaea *Pyrococcus horikoshii* and *P. furiosus* have GH family 5 (EGPh) and 12 endocellulase (EGPf), respectively. Both enzymes showed different substrate specificity. The crystal structures of the two hyperthermophilic endocellulases have been determined [1, 2]. Especially, the structure of EGPf was solved at an atomic resolution of 1.07 Å [2]. The construction of the system for high amount of production of these enzymes is important for their application to the biomass saccharification process. The expression and preparation of small amount of enzymes of archaea have been carried out by the well-known expression systems of *E.coli.* On the other hand, *Talaromyces cellulolyticus* (formerly known as *Acremonium cellulolyticus*) isolated by Yamanobe et al. [3, 4] in 1982 is one of the high cellulolytic enzyme-producing fungi. Fujii et al. [5] reported that the culture supernatant of *T. cellulolyticus* exhibited a higher cellulase activity and glucose yield from lignocellulosic materials than that of *Trichoderma reesei*. Using the homologous expression system under the control of a glucoamylase promoter of *T. cellulolyticus,* some kinds of cellulases were expressed by *T. cellulolyticus* [6]. *T. cellulolyticus* exhibits the potential ability for high amount of production of enzymes. However, there is no report for the expression of enzymes of archaea using fungi. In this study, we try to express two kinds of hyperthermophilic β-1,4 endocellulases (endo-type cellulase; EGPh and EGPf) from *Pyrococcus* using the expression system of *T. cellulolyticus* [6].

## Materials and methods

Carboxymethylcellulose (CMC) was purchased from Sigma–Aldrich. All other chemicals were of the highest grade commercially available.

### Gene optimization and construction of expression vectors of EGPh and EGPf

**Fig. 1:**
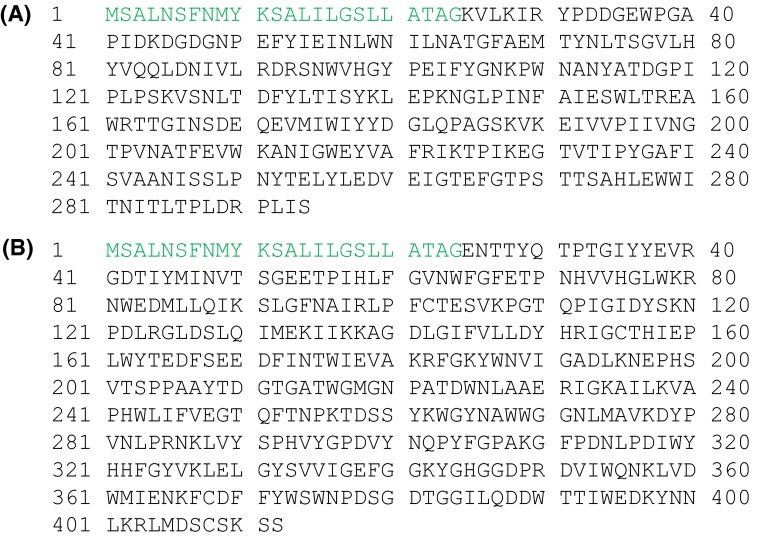
Amino acid sequences of EGPf and EGPh. **a** Amino acid sequence of EGPf **b** Amino acid sequence of EGPh. *Green* secretion signal sequence of Cel7A from *T. cellulolyticus*

**Fig. 2:**
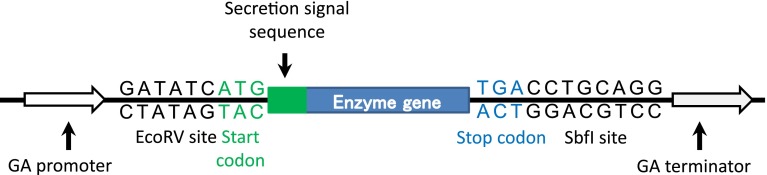
Overview of expression cassette region pANC202. The 1.4 kb promoter region and 0.4 kb terminator region from *glaA*. Endocellulase genes with Cel7A secretion signal sequence were incorporated by *Eco*RV/*Sbf*I site of pANC202 expression vector

Constructions of hyperthermophilic endocellulase expression vectors for *T. cellulolyticus* were performed as described previously [6]. The genes of EGPh and EGPf are containing their signal sequences of *Pyrococcus sp.* [1, 2]. Therefore, their signal sequences were deleted and their codon usage was optimized for *T. cellulolyticus* for the construction of expression vectors. Codon usage for *T. cellulolyticus* was calculated using ORF data of *T. cellulolyticus* genome data (unpublished data). Table 1 shows the codon table indicating the most frequently used codon in *T. cellulolyticus.* The EGPh and EGPf gene were optimized for *T. cellulolyticus* using Table 1. The optimized genes of EGPf (1-270 amino acid) and EGPh (1-388 amino acid) were synthesized (de novo gene synthesis) and assembled by GeneArt gene synthesis service (Life Technologies, Tokyo, Japan). For secretion, putative secretion signal sequence (MSALNSFNMYKSALILGSLLATAG) of *T. cellulolyticus* Cel7A was fused to N-terminal of endocellulase genes (Fig. 1). Synthesized EGPf and EGPh genes contain *Eco*RV site at N-terminal and *Sbf*I site at C-terminal, respectively. The expression plasmids pANC236 for EGPf and pANC237 for EGPh were constructed by introduction of synthesized gene part digested with *Eco*RV/*Sbf*I into the *Eco*RV/*Sbf*I site of pANC202 containing a glucoamylase (*glaA*) promoter and terminator (Fig. 2). All ligated gene fragments and their ligation sites were verified by DNA sequencing.

**Table 1:** The most frequently used codon for each amino acid residue in *T. cellulolyticus* is shown

F	TTC	S	TCT	H	CAT	E	GAA
L	CTC	P	CCT	Q	CAA	C	TGC
I	ATC	T	ACC	N	AAC	W	TGG
M	ATG	A	GCT	K	AAG	R	CGA
V	GTC	Y	TAC	D	GAT	G	GGC
						Term	TGA

### Homologous expression of EGPf and EGPh

Protoplasts of *T. cellulolyticus* YP-4, which is a uracil auxotrophic strain derived from *T. cellulolyticus* Y-94 (CBS 136886, FERM BP-5826), were transformed with pANC236 and pANC237 by nonhomologous integration into the host chromosomal DNA, respectively [7]. Gene integration into prototrophic transformants was verified by genomic PCR. Chromosomal DNA of the transformants was purified using the Gentra Puregene Yeast/Bact. Kit (Qiagen, Valencia, CA, USA). EGPf and EGPh were secreted into culture supernatant of Y236 (YP-4 transformed with pANC236) and Y237 (YP-4 transformed with pANC237) grown in medium containing 20 g/L soluble starch (Wako Pure Chemical Industries, Osaka, Japan) and 2.0 g/L urea as described previously [6].

### Enzyme purification

The culture supernatant containing hyperthermophilic endocellulase was subjected to heat treatment (70 °C for 10 min). The denatured protein was removed by filtration (using 0.22 μm filter). The culture filtrate was desalted on an AKTA purifier (GE Healthcare, Buckinghamshire, UK) using a HiPrep 26/10 Desalting column (GE Healthcare) equilibrated with 20 mM 2-(N-morpholino) ethanesulfonic acid buffer, pH 6.5, containing 0.01 % NaN_3_. The desalted sample was applied to a Resource Q column (6 ml, GE Healthcare) equilibrated with the same buffer. The active fractions were pooled and concentrated by a Vivaspin 20 concentrator (10,000 MWCO, Sartorius AG, Goettingen, Germany), dialyzed against 20 mM sodium acetate buffer (pH 5.0), and stored at 4 °C until use. The purity and size of the protein was analyzed by SDS-PAGE using precast NuPAGE 4–12 % polyacrylamide Bis–Tris gels (Life Technologies).

### Enzyme and protein assay of EGPf and EGPh

Endocellulase activity was measured in 50 mM sodium acetate buffer (pH 5.0) containing 1 % CMC for 1 h. Thermoactivity was measured at temperature ranging from 50 to 80 °C for 1 h. The released reducing sugars were measured by the dinitrosalicylic (DNS) acid assay [8]. Protein concentration was determined by the Pierce BCA Protein Assay Kit (Pierce) using bovine serum albumin as the standard. The samples were dialyzed against 20 mM sodium acetate buffer (pH 5.0) for 2 h before measurement of protein concentration.

## Results and discussion

### Expression of EGPf and EGPh in *T. cellulolyticus*

**Fig. 3:**
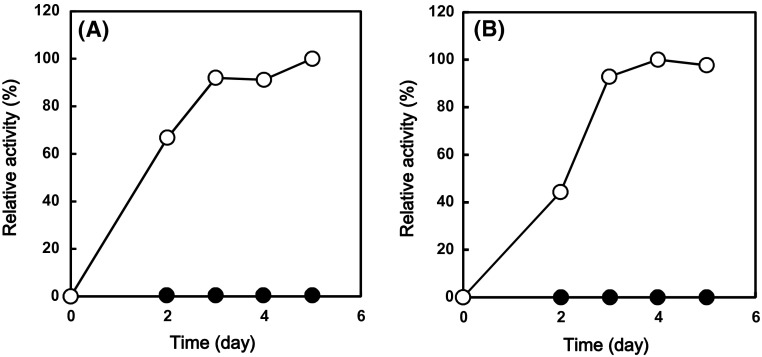
Time course of the expressed activity for the recombinant endocellulases (**a** EGPh and **b** EGPf). The hydrolytic activity of the enzymes toward CMC from the *T. cellulolyticus* transformed with the plasmids pANC236 (EGPf) and pANC237 (EGPh) (*open circle*) and the original strain *T. cellulolyticus*Y-94 (*filled circle*) was measured by DNS [8] at pH 5.0 (20 mM sodium acetate buffer) and 80 °C

**Fig. 4:**
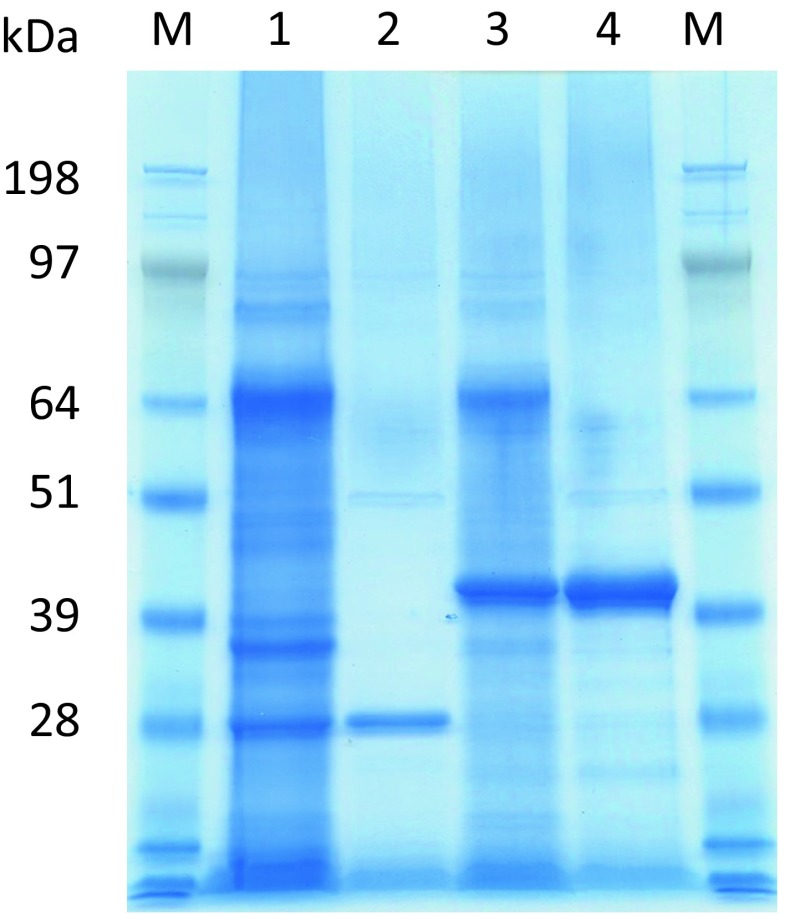
SDS-PAGE of EGPf and EGPh. *Lanes* M molecular weight marker, *1* culture supernatant of Y236 (containing EGPf), *2* culture supernatant of Y236 (heat treated, 70 °C for 10 min), *3* culture supernatant of Y237 (containing EGPh), *4* culture supernatant of Y237 (heat treated, 70 °C for 10 min)

Hyperthermostability of archaeal cellulases is important for industrial purpose such as saccharification of cellulolytic biomass at high temperature. Some of the recombinant enzymes can be prepared by the expression system of *E. coli* and have been characterized [1, 2, 8, 9]. For their industrial purpose, the ability for the high amount of the enzyme production by filamentous fungi has been expected. It has been reported that some valuable proteins derived from bacteria or eukarya could be expressed using filamentous fungi [9]. However, there is no report about the expression of archaeal enzyme by filamentous fungi. Therefore, we tried to express the hyperthermophilic cellulase genes of *Pyrococcus sp*. using our expression system of filamentous fungus *T. cellulolyticus* [6]. The homologous expression system of *T. cellulolyticus* was constructed to produce the target recombinant cellulolytic enzymes without the production of variety of cellulolytic enzymes. Thus, codon optimization was applied to archaeal protein expression. From ORF data of *T. cellulolyticus* genome (unpublished data), codon table optimized for *T. cellulolyticus* was constructed (Table 1). For the two archaeal hyperthermophilic endocellulases, the codon usages for EGPf (1–270 amino acid) and EGPh (1–388 amino acid) genes (Fig. 1) was optimized for *T. cellulolyticus* using Table 1 and synthesized by de novo gene synthesis service. For the extracellular secretion, the signal sequences of the individual archaeal endocellulases were removed and the signal sequences of Cel7A from *T. cellulolyticus* were fused at N-terminus of these genes (Fig. 1) and the expression was controlled by GA promoter (Fig. 2). *T. cellulolyticus* was transformed with the plasmids pANC236 (EGPf) and pANC237 (EGPh) by nonhomologous integration into the host chromosomal DNA [7], and the endocellulase was expressed by starch induction [6]. The expressed endocellulases in the culture supernatant were analyzed by the hydrolytic activity toward CMC, as described in materials and method. Figure 3 shows the time course of the expressed activity for the recombinant endocellulases. For 3–5 day, the expression levels reached the saturation. The expressed proteins in the culture supernatant were analyzed by SDS-PAGE. Figure 4 shows the SDS-PAGE of the culture supernatant after the induction for 5 days with and without the heat treatment. The proteins derived from *T. cellulolyticus* were mostly removed by heat treatment (70 °C for 10 min). After the heat treatment, major bands corresponding to the molecular weight of 28 kDa and 40 kDa were observed (Fig. 4). It is reported that EGPh and EGPf contain the membrane binding region at C-terminus and the region with unknown function at N-terminus, respectively. By removing the regions, the expression of the recombinant enzymes succeeded in *E.coli* [1, 2]. Similar results were obtained in the case of *T. cellulolyticus.* Furthermore, significant activities of EGPf (28 kDa) and EGPh (40 kDa) were also observed. After heat treatment, protein concentrations of the dialyzed samples were measured by BCA protein assay kit. The protein concentrations of EGPf and EGPh expressed estimated to be 0.63 and 0.80 mg/ml, respectively.

### Thermostability of EGPf and EGPh

**Fig. 5:**
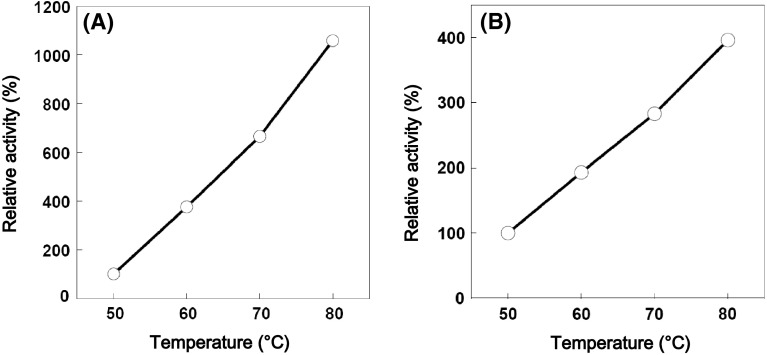
Thermostabilities of endocellulases. **a** Relative activity of EGPf, **b** Relative activity of EGPh

After the purification, the temperature dependence of EGPf and EGPh was examined by measuring the hydrolytic activity using 1 % CMC as substrate in 50 mM sodium acetate buffer (pH 5.0). As shown in Fig. 5, relative activities of EGPf and EGPh were increased ranging from 50 to 80 °C. This result indicates that these *T. cellulolyticus* expressed EGPf and EGPh exhibiting highly thermostability. The recombinant EGPf and EGPh prepared by *E. coli* showed the similar temperature profiles [10, 11]. Furthermore, it was clarified that the activity was not decreased after heating at 85 °C for 2–3 h (data not shown). Thus, this is the clear evidence that hyperthermophilic endocellulases of archaea are expressed by *T. cellulolyticus* and their characteristics are almost same as those prepared by *E. coli.*

In this research, glucoamylase promoter was used for starch induction. In *T. cellulolyticus,* however, there are some promoters which are stronger than glucoamylase promoter. To improve the yield of recombinant protein, the combination of the strong promoter and signal sequence, and optimized culture condition seem to be important. This is the first step for industrial scale production of archaeal protein using filamentous fungi.
